# BITES study: A qualitative analysis among emergency medicine physicians on snake envenomation management practices

**DOI:** 10.1371/journal.pone.0262215

**Published:** 2022-01-07

**Authors:** Anna Tupetz, Loren K. Barcenas, Ashley J. Phillips, Joao Ricardo Nickenig Vissoci, Charles J. Gerardo

**Affiliations:** 1 Department of Surgery, Duke University School of Medicine, Durham, North Carolina, United States of America; 2 Duke Global Health Institute, Duke University, Durham, North Carolina, United States of America; Hong Kong Polytechnic University, HONG KONG

## Abstract

**Introduction:**

Antivenom is currently considered standard treatment across the full spectrum of severity for snake envenomation in the United States. Although safe and effective antivenoms exist, their use in clinical practice is not universal.

**Objective:**

This study explored physicians’ perceptions of antivenom use and experience with snake envenomation treatment in order to identify factors that influence treatment decisions and willingness to administer.

**Methods:**

We conducted a qualitative study including in-depth interviews via online video conferencing with physicians practicing in emergency departments across the United States. Participants were selected based on purposive sampling methods. Data analysis followed inductive strategies, conducted by two researchers. The codebook and findings were discussed within the research team.

**Findings:**

Sixteen in-depth interviews with physicians from nine states across the US were conducted. The participants’ specialties include emergency medicine (EM), pediatric EM, and toxicology. The experience of treating snakebites ranged from only didactic education to having treated over 100 cases. Emergent themes for this manuscript from the interview data included perceptions of antivenom, willingness to administer antivenom and influencing factors to antivenom usage. Overall, cost-related concerns were a major barrier to antivenom administration, especially in cases where the indications and effectiveness did not clearly outweigh the potential financial burden on the patient in non-life- or limb-threatening cases. The potential to decrease recovery time and long-term functional impairments was not commonly reported by participants as an indication for antivenom. In addition, level of exposure and perceived competence, based on prior education and clinical experience, further impacted the decision to treat. Resources such as Poison Center Call lines were well received and commonly used to guide the treatment plan. The need for better clinical guidelines and updated treatment algorithms with clinical and measurable indicators was stated to help the decision-making process, especially among those with low exposure to snake envenomation patients.

**Conclusions:**

A major barrier to physician use of antivenom is a concern about cost, cost transparency and cost–benefit for the patients. Those concerns, in addition to the varying degrees of awareness of potential long-term benefits, further influence inconsistent clinical treatment practices.

## Introduction

The WHO estimates a global yearly count of 2.7 million snake envenomations that cause up to 138,000 fatalities and approximately 400,000 amputations and permanent disabilities [1]. While endemic venomous snakebites in the US are rarely fatal, with a reported 5 deaths out of 8,000 snake envenomations, the CDC acknowledges the fatality rate is higher with reduced access to high-quality medical care. Medical care is imperative to keeping the mortality rate low and to limit disability and loss of function. In fact, the rate of permanent disabilities in the US following rattlesnake envenomations is estimated to be as high as 44% [2].

Antivenom therapy is used to reduce inflammation, necrosis, hypotension, defibrinogenation, thrombocytopenia and neurotoxicity caused by snake venom [3–8]. Recent studies have found that patients who were treated with Crotalidae polyvalent immune fab [ovine] antivenom (FabAV) had better functional outcomes 14 days after envenomation than those who received placebo [9]. Subgroup analysis showed that those treated earlier had a faster recovery than those who had a delay to care [10]. Associated risks of using FabAV include hypersensitivity and serum sickness, which are both infrequent and tend to be mild, making it a low-risk treatment [11]. The other available US antivenom, Crotalidae immune F(ab’)_2_ [equine] antivenom (F(ab’)_2_AV) has similar rates of adverse reactions in the initial comparison trial [7]. Despite this evidence, there remains wide variability in clinicians’ approach to treating snakebite patients, especially among copperhead snakebites [12, 13].

To further improve medical care for patients, we must better understand the factors influencing snakebite treatment and decision-making by physicians to better address disability and loss of function experienced by snakebite patients. Therefore, this qualitative study ‘BITES: Beliefs Influencing Treatment in Snake Envenomation Survivors’ explores physicians’ perceptions of antivenom use and experience with snake envenomation management to identify factors that influence treatment decisions and willingness to administer.

## Methods

### Ethics statement

This study has been approved by the Duke Health Institutional Review Board, Protocol Number: Pro00103272.

### Study design

We conducted an exploratory qualitative descriptive study following, using semi-structured in-depth interviews and an inductive thematic analysis approach [14]. Recruitment began in January 2020, and interviews took place from January to March 2020. Data analysis continued through June 2020.

### Research team and reflexivity

#### Personal characteristics

Two female research assistants conducted the interviews. Both interviewers have Master of Science degrees and are trained in qualitative research methods. The study team included a physician with extensive snakebite research experience, a licensed physical therapist, PhD and data specialist, as well as a clinical research expert with a Master in Public Health.

#### Relationship with participants

The interviewers had no prior relationships with any participants. All participants were first contacted by the interviewers via email with a study-specific email address to invite them to participate and schedule an interview date. The interviewers introduced themselves personally to the participants at the beginning of the interview and provided a short background on their role in this study.

### Recruitment

We aimed to include physicians working in emergency departments (EDs) across the US, regardless of specialty or level of experience treating snakebites. We only included physicians who have completed residency programs. We included physicians working in academic, teaching and community hospitals. We used a snowballing technique to reach a population difficult to contact directly [15]. We first queried current emergency physicians at our institution for contact information of colleagues at other institutions across the US who may be interested in participating. We asked those who participated in our study to recommend any other colleagues who might be willing to be interviewed as well. We enrolled new participants until qualitative data collection reached thematic saturation and the study team decided to have gathered a representative sample based on snakebite management experience, years of experience and workplace setting. To determine data saturation and variation of the study sample, the interview transcripts and main topics emerging in each interview after the first round of 6 interviews were discussed between the two data analysts before moving forward with data collection. We completed data collection after 16 interviews.

### Interview procedure

Physicians implied consent by scheduling an interview, as communicated to them in the invitation email. Interviews were conducted through video conference from a private room on University premises and the audio recordings were stored on a secure cloud-based server. Typically, each interview lasted about 30–45 minutes, though some interviews lasted longer depending on participant availability and length of answers. Each interview was guided by a pre-defined, semi-structured interview guide that was piloted with emergency physicians to evaluate the comprehension and adequacy of the questions. No repeat interviews were conducted. All interviews were first machine-transcribed and then edited for accuracy by research assistants. The research assistants prepared brief field notes after each interview that served as a basis for discussion with the other interviewers during the data collection process.

The transcripts were sent back to participants for review and approval.

### Data analysis

Data were analyzed through an inductive content analysis by the two interviewers, based in an idiographic approach. The emergent codes were organized into themes based on conceptual similarities, which represent the qualitative information gathered: The two analysists first separately performed open coding, then created axial codes. The axial codes were discussed, and a selective coding approach generated the codebook. The codebook was developed based on the first four transcripts and adapted iteratively as new data emerged throughout the analysis process. The full coding scheme is available in S1 File. All transcripts were independently coded by two investigators using a common codebook in Nvivo 12 software [16]. Then, investigators cross-validated the results by discussing the codes and themes of each interview to reach a consensus. The analysts then jointly created analytic memos based on the emergent themes, that served as a basis for discussion with the rest of the study team. All participants received a presentation of the emergent themes and preliminary results to validate the content and ensure accuracy of interpretation.

## Findings

For this manuscript, we use a selection of the emergent codes that were analyzed and grouped into open, axial, and selected codes (S1 File). The available codes were grouped further into the following themes for this manuscript and the specific study objectives: perceptions of antivenom, willingness to administer and influencing factors to administer antivenom. Table 1 outlines emergent themes and codes used for this analysis.

**Table 1:** Emergent themes and codes.

Theme	*Codes*
**Perceptions of antivenom**	*Indications and effectiveness*
	*Risks and side effects*
**Willingness to administer antivenom**	*Treatment hesitancy and perceived confidence*
**Influencing factors**	*Availability/accessibility*
	*Prior education of EM residents in snakebite management*
	*Cost*
	*Usage and perceptions on available resources*
	*The role of scientific evidence and general suggestions to improve patient care*

Participant quotes will be identified by indicating the Participant numbers (eg. P1).

### Participant characteristics

A total of 69 physicians were invited to participate in this study, 29 responded to the email invitation, 7 declined participation. Another 6 participants were lost to follow up after providing initial consent. This study included a total of 16 physicians working in EDs, the majority identifying as male (n = 14 and trained by an emergency medicine residency program (n = 15). One quarter of respondents completed a fellowship in clinical toxicology. The years of clinical experience as well as numbers of snakebites treated were fairly evenly distributed. Six participants practiced in North Carolina, followed by California (n = 2), Missouri (n = 2), New York (n = 2), Florida, Michigan, New Mexico and Texas (Table 2).

**Table 2:** Participant characteristics.

**Sex,** *n*	Male	14
	Female	2
**Age,** *Median (IQR)*	--	43 (37.3, 51.3)
**Residency,** *n*	Emergency Medicine	15
	Pediatrics	1
**Fellowship,** *n*	Toxicology	4
	Pediatric Emergency Medicine	2
	Other*	2
	None	8
**Years of Experience**	0–10 years	6
	11–20 years	5
	> 20 years	5
**Snakebites treated**	0	2
	1–10	5
	11–50	5
	51–100	2
	> 100	2
**Hospital Setting**	Academic	7
	Community	5
	Teaching	4
**Hospital Region**	Suburban	8
	Urban	6
	Rural	2
**State,** *n*	NC	6
	CA	2
	MO	2
	NY	2
	Other***	4
**ED Volume annual,** *range*	--	12,000–242,000

* Hyperbaric Medicine, Global Health Emergency Care.

** Academic: Medical school and faculty/ academic research institution onsite

Teaching hospital: University-affiliated facility to teach students and residents, but no medical school onsite; Community: no affiliation with academic institution.

*** FL, MI, NM, TX.

### Perceptions on antivenom

#### Indications and effectiveness

According to participants, antivenom use would be indicated by laboratory abnormalities, progression of swelling (especially across joint lines), systemic toxicity, coagulopathy, compartment syndrome, widespread ecchymosis, signs of tissue damage, changes in hematologic status and if symptoms severely impacted mobility. Antivenom was reported to always be indicated if there was a perceived risk of losing life or limb. Generally, the greater the number of bites and level of perceived dysfunction based on the bite location, the more likely antivenom is to be administered. One participant said, *“Correct*. *Um*, *I mean I think*, *I think it has the potential to be beneficial*, *[INT*: *Uh huh] in the right case*, *but it’s not something that I would give to every copperhead [bite patient] just because they got bit by a snake*, *because I don’t think that the indication is there for every copperhead without the right symptoms*.*” P13*

Participants explained that antivenom would not be indicated for dry bites or patients with no signs of envenomation. In less severe cases with mild swelling or a minimal envenomation syndrome, most physicians agreed that observation and routine supportive care would be sufficient. While one participant specifically pointed out that, in her opinion, pain alone was not a sufficient indication for administering antivenom, others mentioned that antivenom is effective in controlling pain. One participant mentioned that antivenom use in snakebite patients could limit opioid prescriptions.

Effectiveness of antivenom treatment was believed to vary between patients, depending on their underlying health conditions, the time to treatment and complicating factors that would cause their envenomation to be more severe. Antivenom was perceived as being very effective for decreasing swelling and swelling-related pain and tissue damage. Those more familiar with the snakebite treatment literature mentioned decreased morbidity and faster return to function with antivenom; however, there was no overall consensus among participants if those potential benefits would be significant enough to indicate antivenom use for milder envenomations. One participant mentions: *“So*, *I typically claim that if*, *you’re having extensive*, *significant tissue damage or*, *tissue damage that seems to be progressive*, *we typically would recommend [fab antivenom] to kind of help decrease*, *disability down the road*, *disability and pain later on*. *So that’s kind of how we discuss this I guess*, *would be our recommendation*.*” P15*

Several participants were uncertain of the potential benefits or effectiveness of antivenom due to lack of personal experience and were unaware of any potential improvement in long-term outcomes: *“[M]y impression is that [antivenom] is just for […] halt[ing] the progression of the disease […] preventing it from getting worse and stuff*. *But I*, *I don’t know what effects some of the stuff [has] later down the line*.*” P16*

#### Risks and side effects

The vast majority of participants mentioned allergic reactions, including hives and itching, as the main side effect of antivenom; however, they perceived the administration of antivenom to be safe and low risk. Other potential risks included serum sickness and hypersensitivity.

### Willingness to administer antivenom

#### Treatment hesitancy by the providers

While risks and side effects did not seem to be strong barriers to antivenom treatment, the majority of physicians reported being generally hesitant to administer antivenom to their patients. The threshold at which physicians decided to treat with antivenom seemed to be influenced by personal practice and individual risk tolerance. However, potential risks or side effects did not contribute to treatment hesitancy:

*“Maybe it is that I’ve spent a little more time reading about it and stuff, and so I try not to over-treat. So maybe that’s part of it: the toxicology training and the extra reading. Maybe another part of it is I’m aware of the price to some extent. I think that’s much lesser of a reason. Yeah. […] And then, you know, lastly, it’s more of a personal question on practice and stuff like that. I mean my risk tolerance is different than someone else’s risk tolerance.” P16*

Rather, lack of experience in treating snakebite patients may either lead to hesitancy to treat to avoid unknown risks associated with the treatment or to early treatment with fewer indications to reduce the risk of progression of symptoms. Some participants expressed that increasing confidence and perceived competence in snakebite management required personal and practical experience through, for example, being trained in high-prevalence areas, while reading the available literature alone would not be sufficient. Among our interviewees, those with more clinical and snakebite treatment experience generally felt more comfortable withholding antivenom to avoid what they saw as unnecessary treatment. More experienced physicians trained with fewer resources would rely more heavily on clinical judgement.

*“But I think the more experience you have with it, probably the more comfortable you are withholding antivenom than if someone has minimal symptoms. Because if people see any symptoms, they… it’s a strong word, but they kind of panic and they shoot for the antivenom. A lot of times that that’s appropriate. Right? Because that patient would have gotten sicker. But you never know because they’ve gotten antivenom. So would they have gotten better on their own? You know, some people aren’t willing to wait and watch.” P9*

Those with experience using unfractionated antibody antivenoms, which are no longer in use, usually tried to refrain from antivenom use in general. Unfractionated antibody antivenoms had much worse side effects and the majority of their patients eventually recovered. Even with the newer forms available that are safe and low-risk, such practitioners do not view antivenom as vital for the care of mild cases. When it comes to venomous exotic snakes or severe copperhead bites, there was no hesitancy to treat in order to save life or limb.

Institutions without an institutional treatment protocol generally had physicians with differing opinions on treatment plans and more treatment hesitancy. Even those that did have institutional guidelines mentioned room for personal interpretation of the guidelines, as the language was typically not precise enough with clearly defined terms and thresholds (for example, what the medical definition of ‘mild’ swelling is).

One physician, however, mentioned that, based on available data, his institution tends to treat snakebites more aggressively with antivenom than other medical professionals might:

*“But we’ve always, […] based on the data, the decreasing morbidity and […] improving functional outcomes, improving pain long term, I think we tend to be more aggressive than some.” P15*

Other factors contributing to treatment hesitancy included skepticism of scientific data supporting antivenom for non-life-threatening conditions based on funding sources of studies, and the belief that financial costs to the patient would potentially outweigh the clinical benefit of receiving antivenom treatment. Emergency physicians typically did not have the opportunity to follow up with their patients to gather anecdotal evidence, so they reported the absence of an intuitive sense for how well or poorly patients recover and their long-term outcomes. The perceived value of anecdotal experience was demonstrated by one participant who did not recommend antivenom to a neighbor, who later said that his chronic pain after the bite was so bad that he wished to have been treated with antivenom if insurance covered it.

Seeing how the prolonged symptoms impacted his social and work life gave the physician a new perspective on treating snake envenomation patients. After that experience, he saw the value in receiving follow-up data, saying that this information could help physicians gain more confidence in their treatment decisions and shared decision-making:

*“I think [feedback on recovery from discharged snakebite patients] would [help.] And it would be part of my shared decision-making speech with the patient. [INT: OK] And I think that it could potentially cut, you know, as with all shared decision-making—It’s never completely neutral. And so, I think […] it would potentially push me to encourage the patient to use the treatment if there was something where cost was a satisfactory part of the consideration.” P3*

### Influencing factors in the choice for or against antivenom

#### Availability and accessibility

The availability and accessibility of antivenom was not cited as a major concern for treating snakebite patients within our study sample. One participant states:

*“[fab antivenom] was the only available antivenom and is still the only available antivenom here in our institution.” P10*

Other potential barriers for optimal treatment were identified. In some cases, antivenom was not kept in stock at the facility, requiring transfer of either the patient or the antivenom. In such cases, distance, mode of available transportation, and road or weather conditions could impact timely access to care. Access to institutions with available antivenom and experts to treat snakebite patients may be limited due to small clinics, which are not part of larger networks, not being aware of any nearby expert centers, and lack of awareness where to search for referral centers. Accessing antivenom for exotic snakebites could be a challenge depending on the snake type if a local institution or zoo does not have any in stock, and it might have to be delivered from distant locations for very rare bites.

#### Prior education of EM residents in snakebite management

The level of didactic training received during EM residency did not seem to shape the general acceptance of antivenom, but more so the clinical approaches of local experts and mentors during residency. In areas with little to no snakebite patients, the education mainly consisted of didactic training, as well as how to use available resources like the Poison Center Call line and under what circumstances to refer patients.

*“Well, you know, it’s one of those things that all emergency physicians need to know about. I think you’re going to find it a very variable fund of knowledge. And it’s just because with rare exception, they just don’t see that many of them. […] one of the wonderful things about emergency medicine is you have this diversity of things that you get to do. The curse is that you can’t be the world’s expert on everything.”P9*

#### Cost

All interviewees agreed that if administering antivenom would be a lifesaving treatment, cost would not be an influencing factor in their decision-making. However, cost would become an influencing factor when antivenom was used to prevent tissue damage in non-life-threatening conditions. Physicians typically informally weighed the costs and benefits of antivenom in these situations, with the caveat that those who primarily only treat severely toxic bites usually do not consider the cost of antivenom. One physician explicitly named the cost of the antivenom to be a risk factor to take into account.

When it comes to the transparency of the cost of antivenom itself, most were not aware of the exact costs per vial for the hospital to acquire it, as well as for the patient to receive it. Those who were more acutely aware of the pricing had made a deliberate effort to find the information, and sometimes those who did still could not obtain a clear answer. There was uncertainty regarding national standard pricing, a lack of transparency within hospitals, and further uncertainty when it comes to how much insurance may cover.

*“The other consideration for maintenance vials is that they’re super expensive. So a single vial of [fab antivenom] can cost the hospital between three and four thousand dollars. And depending on the charge master and what the hospital wants to charge [a] patient with or without insurance, that could go up, you know, seven times upwards to twenty thousand dollars per vial. So, if you’re giving 6 additional vials, for somebody, you’re looking at about one hundred twenty thousand dollars in cost to patients.” P10*

Despite this uncertainty, physicians were aware that the financial cost was high, and patients may be partly or fully responsible for covering it. In one case, however, the physician doubted that patients would actually be charged by the hospital if uninsured, stating: *“I just*, *I highly doubt that any of the ER patients I see are actually receiving bills*, *like*, *I don’t know*.*” P 16*

Cost emerged as the biggest barrier to antivenom treatment. Some participants expressed that, if costs were minimal, they would be more likely to treat more aggressively in mild cases to decrease chronic morbidity. However, some maintained that they still did not see mild cases as being an indication for antivenom, no matter the cost:

*“I think having greater availability of antivenom at lower cost can take the question of whether or not to give antivenom to patients, in the equation that anyone with minimal symptoms may be able to get the antivenom without just thinking about how it will affect them financially. I think it’s going to be huge.”P10*

#### Usage and perceptions on available resources

Table 3 provides an overview of the available resources and influencing factors that impacted their utilization.

**Table 3:** Overview of available and utilized resources.

	Available/ Utilized Resources	Influencing factors to utilize resources
**Written/ electronic resources**	Institution’s own treatment guidelines, discharge information (based on CDC, poison center and local experts)	Awareness of existing guidelines
	Apps and online resources (Blogs, antivenom manufacturer’s website, EM associations, general EM resource platforms, discussion boards)	Ranges in specificity of provided information
	Standard textbooks	Adherence to guidelines impacted by clinical judgment and individual patient factors
	Manufacturer’s FDA approved guidelines/ package insert	Decrease of cognitive load
	Related scientific literature	Recency of information
	Topic-related continuing education and conferences	Availability of pediatric specific resources
		Online resources generally deemed useful and acceptable (Some skepticism on quality of online resources; Utilization driven by individual physicians rather than institutions)
**Poison Center Call line**	Reconfirm treatment plan	High availability over the phone or at bedside
	Public health monitoring	Considered high quality information
	Access to written documents for provider and patients	Small nuances in recommendations based on individual consultant
		Source of clarifications for guideline interpretations
**Personal resources/ communication**	Local herpetologist societies and zoos for snake identification	Too little available resources for exotic snakebite management
	Institution’s pharmacists for dosage recommendations and cost information	Possibly available for treatment consultation at bedside, varying availabilities and involvement in patient care
	Local experts and onsite toxicologists	Experts better equipped for determining appropriate management strategies
	Reliance on previous education, residency, fellowship, board preparation, CME	Variations in expertise
	Own published literature	
	Previous mentorships	
	Self-driven interest in topic	
**Scientific evidence**	Published peer reviewed manuscripts	Personal experience may take precedence
	Treatment algorithms	Seldomly used as a discussion tool with patients
		Level of awareness of evidence-based indications on chronic pain and functional long-term outcomes
		Lack of data on management of non-life-threatening conditions
		Lack of knowledge on possible long-term harm of different treatment options
		Level of awareness of current research
		Perceived level of quality of available evidence
		Skepticism towards pharmaceutical company-funded research

While resources seemed to be readily available, some physicians pointed out that clinical judgement and personal experience may take precedence over general guidelines. The Poison Center Call line generally was thought to be a valuable and high-quality resource for physicians at bedside. However, if experts were available within their own institution, the physicians would consult them prior to using Poison Center Call lines. The benefits of Poison Center Call lines were that they were always available over the phone and potentially on bedside, yet one physician who worked for Poison Center Call lines raised the concern that over the phone consultations may result in hesitancy to follow their recommendations by the treating physicians. None of the other study participants who used Poison Center Call lines as a resource shared that concern. However, small nuances in their recommendations could occur based on the individual consultant.

Overall, the available information on toxicology and pathology in the United States was thought to be of high quality and the physicians generally trusted the guidelines, recommendations as well as the safety of antivenom. In terms of antivenom though, some physicians voiced concerns about the trustworthiness of the data behind maintenance vial recommendations in regards of quality and the limited available evidence of its necessity:

*“…the question is, do we have a good enough study that a perfectly defined mild, moderate, severe bite that shows a definition that they should actually benefit in the time that they got back to function. I don’t know that […] we’ve shown enough evidence to show that giving someone body [Fab] will get you back to function faster.” P13*

In addition, some felt that most of the evidence and available guidelines were snake-specific and non-transferrable. In terms of the quality of the available resources, the validity of online resources was questioned by a few participants, and one physician suggests increasing efforts in distributing better information to reach the physicians at bedside.

#### The role of scientific evidence and general suggestions to improve patient care

While scientific literature was, in a few cases, used as a tool to discuss treatment indications with the patients, the physicians also stated that personal experiences and beliefs might take precedence in choosing and recommending treatment options. Very few physicians were aware of studies investigating the effect of antivenom on pain or other long-term functional outcomes. In fact, several physicians reported a lack of awareness of ongoing scientific efforts and advancements in snakebite research and were unaware of available high-quality studies to guide their treatment decisions.

In addition, skepticism of the available data was raised when funded by pharmaceutical companies, voicing the need for different funding sources, as well as skepticism surrounding the quality of available research data supporting antivenom for non-life-threatening conditions.

Table 4 provides an overview of recommendations the physicians provided specifically to enhance the scientific research and literature surrounding snakebite management.

**Table 4:** Recommendations to improve scientific evidence base on snakebite management practices.

**General**	Increase dissemination practices and awareness of research advancements
	Increased efforts to conduct high quality research to support antivenom for non-life-threatening conditions
	High cost of antivenom requires high-quality evidence to base treatment decisions on
**Study design**	Randomized controlled trials (large sample sizes, causality)
	Separate studies for copperhead and rattlesnake envenomation treatments
	International snakebite research in areas with higher morbidity and mortality
**Outcome measures**	Standardized and robust (dosing regimen; timing regimen; specific and measurable definitions of mild, moderate and severe cases)
	More concise clinical endpoints
	Differences in hospitalization rates and length of stays with and without antivenom administration with a focus on associated costs
	Pediatric population: specific research designs with tailored and appropriate outcome measures of pediatric population
**Research aims**	Comparison of different available antivenoms (F(ab’)2 vs Fab) at various dosages to determine the most cost-effective treatment
	Effectiveness of maintenance vials
	Recovery times to avoid relying on personal experiences; antivenom development that covers a broad range of snakes
	Improving available antivenom products
	Ways to effectively administer antivenom as early as possible, identifying effective first aid treatments in the field
	Better understanding the sequelae of snake envenomations
	The effect of antivenom on return to function and long-term outcomes
	Cost-benefit analysis
	Impact of snake envenomation severity on functional outcomes and associated treatment costs

One physician pointed out that, while there were many suggestions on new evidence-based guidelines, we should also seek to understand what keeps treating physicians from following the already existing guidelines and then move forward promoting a socially and fiscally responsible practice:

*“In those cases, where the diagnosis is not in question, nor the treatment in question, but at which time and at which patient this treatment would be appropriate for… Those are when I feel that we need to have more guidance and more standards, and then develop and fine tune those standards over a period of experience.” P5*

Generally, the physicians agreed that to improve patient care, focus should be on high-quality evidence and guidelines, continuing education, patient-friendly information, increased transparency of long-term outcomes for EM physicians, and reassessing the cost for patients. Table 5 provides an overview of the suggestions provided by the study participants.

*“[F]irst of all, education, and trying to find a platform to disseminate it. And that is so that EM-RAP [Emergency Medicine Reviews and Perspectives] is a great way of doing it, number 1. And number 2: I think if there was a very easy website that someone could just [find] snakebite guidelines and […] anybody from anywhere could easily [access], and then it goes through these different tabs so you know, indications, diagnostics, evaluations, patient education, what to notify a patient, and […] that you could easily print out […] and give it to a patient and go over information.” P12*

**Table 5:** Suggestions to improve patient centered clinical best practices in snakebite management.

Areas for improvement	Suggestions
**(Continuing) education on snakebite management**	Educate physicians at bedside when and where to seek guidance an snakebite patients; due to low prevalence every physician should know where to find topic experts and when to reach out
	More emphasis on regularly updating poison center information and available guidelines
	Using different channels of disseminating the available literature through webinars or educational opportunities
	Enforcement of systems that ensure wide information dissemination and awareness of new information by physicians
	Centralized one-stop resource online platform to identify experts for guidance, information resources, referral centers, most recent treatment guidelines, ability to filter the information by specific regions to overcome challenges in the snake identification
	Understand the barriers that keep treating physicians from following the guidelines
	Assess current level of education on snakebites and knowledge among currently practicing physicians
	Educational lecture that reaches wide audience on current snakebite management practices and advancements
**Cost-benefit and access**	Increase efforts directly targeting cost innovation and drug development for non-life-threatening bites
	Increase access to antivenom and medical care globally
**Improve existing and commonly utilized resources to improve care**	Update and educate Poison Centers on the most recent evidence
	Invest in a new easy to use protocol to be followed and shared by Poison Centers
	Promote Poison Center as a resource for patients looking for access to care
**Patient-friendly communication and information sharing**	Scientific data in patient-friendly terms, including advantages and disadvantages on antivenom usage, cost calculations, percentages of patients recovering after different treatment approaches, recovery time
	Promote shared decision-making and make the patient education process more objective across providers
**Implementation of feedback loops to EM physicians**	Hospital-based system that feeds back the treatment outcomes on a case-by-case basis to increase the confidence in treatment choices and tool for shared decision-making
**National guideline and best-practice protocol development**	Existing experts in the field to assess and review all information to create a clinical guideline that would be widely acceptable, while taking some geographic and snake type variations into account
	Decrease cognitive load on physicians
	Specific clinical indicators or scoring system to inform the decision-making process, improve consistency across providers
	Applicable to specific contexts, facilities, and patient demographics
	Education on when to deviate from guideline recommendations
	Regional guidelines, based on local expert opinions
	Symptomatic approach
	Information on timing, appropriateness of treatment, clinical factors, specific recommendations on lab value ranges
	Help manufacturers to better control the supply and demand and ultimately lower the costs of the antivenom

## Discussion

This study details the barriers to antivenom treatment and physicians’ needs in order to improve patient care by utilizing widely accepted best practices. Treatment approaches and perceptions of antivenom usage were influenced by a wide variety of factors in snake envenomation.

Barriers to using antivenom were rooted in a wide variability in experience, awareness, and trust in available resources and evidence to inform physician decision-making. Some participants primarily relied on textbooks, raising questions on the timeliness and inclusion of current advancements in snakebite management. Aside from the Poison Control Center call line, there was little overlap of widely used and accepted resources by our participants. Having such a variability in resources including local expert opinions, on-site toxicologists, websites, apps and blogs increases the challenges to ensure consistent evidence-based recommendations. Participants echoed this notion and called for a systematic and high-quality national guideline, with precise and applicable clinical treatment recommendations. Our sample did not appear to be broadly aware of the detailed national guidelines that already exist [17, 18]. Available scientific data, when funded by pharmaceutical companies, was often met with skepticism by our participants, especially when the findings recommended antivenom for milder cases. The fact that the majority of clinical trials in medicine are funded by industry did not seem to influence this belief [19, 20].

Another consideration the majority of study participants brought up was the potential financial burden for patients, lack of transparency surrounding cost, and the need for cost–benefit analyses regarding initial doses and maintenance vials of antivenom. The potential detrimental financial burden for patients, in other studies referred to as ‘financial toxicity’ [21–23], is a known factor among medical practitioners in the United States. However, current research on cost transparency focuses on the hesitancy of seeking care from the patient’s perspectives [24, 25], but not on how cost transparency and deeming financial toxicity for the patients can influence the provider’s treatment approaches. Our study demonstrates how cost is an important factor that providers consider when advising patients on snakebite envenomation treatment options.

In addition to the possible financial burden influencing decision-making processes, some physicians based their clinical decision-making on their clinical experiences and conversations with colleagues and mentors, instead of current scientific evidence. Potential reasoning behind the experience-based medicine approach, instead of evidence-based [26], was the lack of trust in the data, as well the perceived superior value of clinical experience and competence. Providers, as well as patients, tend to be hesitant in accepting treatment suggestions based on poorly designed studies, increasing the value of expert opinions in the decision-making process [27]. Clinical judgment is a cornerstone of clinical practice to interpret clinical data. However, “like any judgment, these perceptions are not always reliable. It is known that physicians are highly variable in their interpretation of clinical data. […] Further, they disagree with themselves when presented with the same information at two points in time” [28]. Other influencing factors in clinical decision-making may also include autonomy, education, understanding the patient status and awareness of the situation. Another challenge is the successful translation from research findings into clinical practice. Grimshaw et al. emphasize the importance of synthesizing research findings of specific topics to facilitate the integration in clinical practice. An assessment of barriers and facilities among different groups and settings is deemed critical to identify opportunities for successful knowledge translation into clinical practice patterns [29].

Lastly, we have found perceived safety and accessibility of antivenom were not considered barriers to treating snakebite patients with antivenom. Given the history of antivenom and strong side effects of the early forms of treatment, it would have not been surprising if some participants, especially those who were trained when the older equine whole immunoglobulin antivenom was available, to base their reservations on antivenom usage on the perceived high risks for patients. While there is still a debate on “whether antivenom manufacturers are producing high quality and efficacious antivenoms” [30], especially in rural tropical areas with the highest burden of venomous snake bites [31], our US study sample perceived the quality and safety of the available antivenoms as high. Copperhead snakebites were generally not considered a life-threatening condition requiring immediate antivenom treatment. This was felt to provide additional time to determine if antivenom is necessary, despite evidence that copperhead snakebite is likely a time-dependent disease [10].

### Limitations

Some limitations to the current study exist, though measures were taken to minimize their effect on the quality of the study. The Principal Investigator was known to some of our study participants, which could have influenced their participation in this study. In order to control for that, we informed the participants that the interviews will be de-identified and sent out for their approval before the PI would have access to the data. In addition to that, the majority of our study sample practiced in North Carolina and academic or teaching hospitals, limiting the representation across the US and community hospital providers.

### Conclusion

The lack of awareness and trust in available scientific evidence regarding the benefits and indications for antivenom especially in non-life-threatening conditions led to a wide variability in treatment approaches by practicing physicians. In addition, the lack of cost transparency further contributed to hesitancy among providers in their treatment approaches. Our study emphasized the need for a widely accepted best practice guideline that is evidence based, includes concise clinical indicators developed by topic experts, and is implemented by practicing physicians.

## Supporting information

S1 Checklist COREQ (COnsolidated criteria for REporting Qualitative research) checklist. (PDF)

S1 File Full coding overview. (DOCX)

S1 Scheme (DOCX)
