# Supplementary material for: BITES study: A qualitative analysis among emergency medicine physicians on snake envenomation management practices
Source: PLoS One. 2022 Jan 7;17(1):e0262215. doi: 10.1371/journal.pone.0262215 (PMC8741014; doi:10.1371/journal.pone.0262215)
Supplement: S1 File — (DOCX) [file pone.0262215.s002.docx]

**BITES study: A qualitative analysis among emergency medicine physicians on Snake Envenomation Management practices**

**S1 File: Full Coding overview and selected quotes**

| **Themes** | Codes | Selected supporting Quotes |
| --- | --- | --- |
| **Snakebite treatment, clinical decision making** | Snakebite management education of trainees | P1:  Yeah. I think maybe once during residency, we had somebody who was bitten by a snake. Uhm… But the rest of the time, it was just all… academic education in preparation for my E.M. boards.  INT: So you mentioned that you were a resident at Z. But now that you work elsewhere, how would you say that the education about snakebite treatment is at your current facility?  P2: Well, I work at a resident, you know resident learning institution. So I think we're a little bit more involved with teaching residents and other attendings about, you know, kind of the oddities like snake bites. But I think our surrounding institutions where they don't have residents and there isn´t ongoing education, I would imagine it's a little bit behind on the data for the educational side. Even at our institution, though, we're still behind. I think just because of the limited amount of snake bites we receive, being that we're kind of in a city that's not very not very good compared to an institution like Z was.  INT: Um alright. So, how much have you been educated on snake bites um through med school, residency, and then I guess on the job training? How – [P5: Gosh] Where did you get your information from?  P5: Uh… I would say that probably all together, maybe five hours worth of CME, my math on snake bites. If you had to put together studying that I had to do for the boards, a talk maybe at a residents conference because there was something neat that somebody said they were travelling somewhere else.  INT: Great, so within your facility uhm, your already said it is a university hospital, so how does the, the training of the residents look like in terms of snake bites?  P13: Um, well all of our ER residents spend a month on toxicology during their residency [INT: Uh huh] So, you know they have that, exposure to us and the Poison Center. And then, you know they all work on the adults and peds side of the ED so they, they get to see snakebites throughout their training [INT: Mhmm], you know. |
|  | Snake identification | P1: Well, if we were to see a victim of a snake bite, first thing we would do is try and identify the snake. [INT: Mm hmm. Right.] Is it poisonous or not poisonous? [INT: Yeah] I think that's sort of critical to the… ah future path that we take in, in terms of care. [INT: mhmm] And If it's not poisonous then we manage the wound and go on our day. [INT: Right]. If it's poisonous, then we have to identify what type to facilitate the antivenom [INT: mhmm] administration at the receiving facility, at at XY.  P2: He was pretty sure it was a black snake and we're pretty sure the black racer based on his description. And we have a snake identifier app on our phone. Most of us do on our phones.  INT: And does that influence your, um, your clinical approach at all - whether the patient knows a) whether it was a snake, definitely that bit them? And b)what type of snake it was?  P3: Um… So I guess, um… um… I guess technically it should. I - I think that, um…. I don't really think it does that much at the end of the day because, you know, I'm – I’m looking at a snake bite in general, like how sick, you know, how bad the wound is, how sick the patient looks. So if it's a significant looking swelling, you know, like that, there is some type of venom - [INT: mhm] I'm presuming based on our epidemiology, or our geography that it did - it would be - if it is a venomous, poisonous snake, it would be a copperhead. So not really. Like it doesn't really influence what we do I think.  P9: Well um, I try and be a good doctor. Um. I try and get the best history I possibly can. Um. You know, there's some key features. Number one is, if you can get a positive I.D. on the snake, that helps a lot. [INT: Mhm] And so if there's an opportunity to… if there's an opportunity to do that, uh either directly with a photograph or by virtue of a description and a location, do that, then, you know, do a good history um and find out what the course of the patient's um… |
|  | Assessment | P15: Um, I think that, I mean, probably somewhere in the middle of that. [INT: OK] I mean I don't know that I personally use a lot of envenomation scores…but certainly if they just have a little mild swelling at the site, I don't think we necessarily would do it then. [INT: OK] If the swelling seems deep or ecchymosis seems to be progressive or passes over a joint, um, so I mean like for example, if the entire foot is edematous and ecchymotic, even if it doesn't cross the ankle, we might still be more likely to, to give [fab antivenom], [INT: mhm] if it’s just a simple bite wound with minimal swelling, we may just watch those and see.  INT: Mhm. And so you mentioned that you also spent some time in a children's hospital. Do you think that the way you approach treating a snakebite patient would be different for a pediatric patient versus an adult patient? P14: I do, only in the way that if they're really young, you know, having them use crutches is really hard. So, a 3 year old using crutches, um, is probably not going to be realistic [INT: mhm] um versus, you know, if it's a 10, a 12 year old, that's someone probably that we can probably train to use crutches. So, if it's really going to be for two weeks that, you know, mom or dad has to carry the kid everywhere they go, they can't walk and they can't use crutches, um, then that kind of changes, you know, the algorithm. With that said, um with copperheads I've had, I have had some families who still just didn't want to give antivenom and were OK, kind of carrying around their kid for [INT: mhm] a week or two until they were able to start bearing weight.  P14: Um, and for them, it was kind of pain and swelling going up their leg. Um, and generally, you know, we’re able to identify that was a copperhead [INT: mhm], so I'm not really worried about systemic toxicity, at least any significant systemic toxicity. So really, it's a matter of getting their pain and swelling under control [INT: mhm], um, which we do with a combination of, NSAIDS, opioids, foot elevation, um, and then depending on how that goes, and I guess just how significant the swelling is, then you could discuss antivenom with them. |
|  | Alternative or supplemental care to antivenom | INT: Mm hmm. So you mentioned if it's available, how would you proceed if it was not available?  P4:I guess it depends on the envenomation, and that's mostly supportive care at that point, depending on if it's a respiratory failure or cardiac failure, kind of a cobra managing symptoms, kind of sympathomimetic access, you know with blood pressure control, heart rate control and ventilatory support and pain management, plus or minus surgical debridement or if there was a significant necrotic area that would require a surgery.  INT: My last question for you is just if you had a patient, what are some of the alternative treatment options if they didn't want to go through with antivenom?  P10: Certainly, pain control monitoring and outpatient follow up, and physical therapy, occupational therapy. Those are all helpful.  INT: Right. OK. So, are there, if you had a snake bite patient and you thought that their symptoms weren't severe enough to warrant using antivenom, then what are some of the alternative treatment options you would do for them?  P15: Usually just observation is the main thing. You know, we watch them for, you know, eh, four hours or so and they won’t have any progression or any signs of significant envenomation, we usually let them go home and come back if that materializes. |
| **Antivenom Treatment** | Effectiveness and indications | INT: OK, great, perfect. And what what are the indications for [fab antivenom]? So, you already mentioned, you know, when you would decide to to administer it. Um, is it pain, swelling, recovery time? What is the expected outcome of using antivenom?  P16: Mm hmm. Um, it’s, uh, increased swelling [INT: Mhm] I think is something that I can objectively quantify. Um, and then, uh, I don't think that pain is, uh, is enough. You know, I think I've had some patients that, hmm, have had pain, uh, increased pain. But I don't think that's pushed me to treat just simply increased pain, [INT: Mhm] Swelling, and, uh, you know, if if there are, um, signs of necrosis, that kind of stuff, those kinds of local effects. Symptoms, I really haven't treated on that much, signs or so. Um, and then certainly we talked about any, uh, signs of like a coagulopathy on on their vascular panels. Again, um, INRs and platelets probably being the biggest thing. Um, and then like I said, I’ve treated one patient with what I thought was a systemic effect; nausea and vomiting. [INT: Yeah, OK, great] Um, so tho those will be my indications for treatment.  P15: But we've always, I think, kind of been, um, you know, based on the data, the decreasing morbidity and decreasing, you know, improving functional outcomes, improving pain long term, I think we tend to be more aggressive than some.  INT: Okay. Great. So how long, how long do you usually give the the patient to kind of observe the bite before you have to make a decision whether antivenom would be indicated or not. Is there a certain time frame [P13: Uhm] that you can..  P13: I think it depends on a little bit where the bite is [INT: Uh huh] Right so. Uhm, you know, a young child that’s like a, a finger bite, I’m going to be more cautious with and and, if it starts to spread faster, more pain that control them I might give them antivenom faster than I would an ankle bite that has more area for swelling and more, uhm, kind of flexibility and that. But [INT: Mhmm] usually if you have significant progression, you know past, past more than one joint uhm, from the bite sight, within like, within an hour [INT: Mhmm] then you know then you start to think, you know, do I need to add antivenom, versus, you know, I think it really depends on the situations because if they had their ankle down to gravity for the past hour of course it’s going to be more painful [INT: Right] so I want to get them in the right positions, get them some pain medicine and you know give them an hour probably to kind of see where we’re going. |
|  | Treatment regimen | INT: And when it comes to the dosage of antivenom treatment, how do you determine that? Is that something that there's a strong protocol for or something that you would have to consult someone else to find out the proper way to do it?  P4: I'd have to, I guess it depends on the antivenom (INT: right) and then I´d have to look it up. I don't have it memorized, so we would probably, at that point, we'd be working with our toxicologists and our emergency pharmacists with the dosing.  P13:  Most of the Copperheads here do not get maintenance vials. And they don’t need them. And so I do not think that it is wise to throw a [inaudible] that doesn’t have indication for why you need 12 vials on somebody [INT: Uh huh] up front on somebody. If you think that they need it, then I think giving them the 4 to 6 bolus dose as previously documented in the literature as being beneficial then, um you know, it can be, can be fine. But most of them don’t even need their maintenance doses so I think that- I think that they are some places that probably, in my opinion, give too much [fab antivenom]. |
|  | Availability and accessibility | INT: Yeah. You said, though, that the antivenom usually is – uh is stored at the facility? Or would that also be with the transportation or getting the antivenom also be another, another factor to consider?  P5: At most of the facilities, it's it's not going to be stored and it would um be one or two things. Um and I would guess which would be the quicker. Could you courier or reverse courier the antivenom to your facility if the patient was able to stay there? But I'm guessing if they were in shock and looking like sepsis and with coagulopathy, the patient probably would be transported almost immediately. And thankfully, we do have a lot of air transport that can help out. [INT: OK] Um. So air transport to a, another facility at least where I'm at is anywhere between a half hour to 90 minute flight time. [INT: Mhm] Um. And then you have to, you know, add in the 15 minutes to warm up to helicopter and a 15 minutes to package a patient. So at best, if from the door I had identified a snakebite concern for an envenomation and shock with coagulopathy and um and called the flight crew right away, I could get somebody packaged up within 20 minutes and then flown to the nearest facility if they had a bed in 30 minutes. [INT: Mhm] So an hour transport time to get somebody the medication if it was available. [INT: Ok. Ok, great.] Well, like I said, I haven't called it up before, nor have I given it.  P4: And I think in our facility there is a limitation because we treat so few toxic snake envenomation, that you know, our hospital doesn't stock it per se, so we're acquiring it anytime there's a toxic envenomation from an outside source, it happens fairly rapidly. But it's not as, you know it happens so infrequently in our institution that, (cuts out for 4 seconds) that´s kind of where we´re at. We just don't treat very many of those.  INT: Okay, got you. Great. And I assume you have, you have the antivenom in stock at your facility?  P13: Yeah. Yes, we do. |
|  | Risk and side effects | INT: Are there any adverse effects of using it?  P8: Well, I mean the patients can develop allergies because this was made out of serum [INT: Mhm] and the new ones are less allergic. But uh I think there’s a big cost issue with [fab antivenom] as well. [INT: mhm]. But that doesn’t really factor that much into your decision to use.  INT: Mhm, yea that's very interesting. So, you mentioned that you think anyone, ideally anyone with minimal symptoms should get antivenom. Is there any concern for any adverse effects or any unintended consequences from an unnecessary treatment?  P10: Yea mostly just the pocketbook consequences, right? The financial cost of it. I think that [fab antvenom] and [fab2 antivenom] are clean enough products that the, um the allergic reaction, the anaphylactic reaction, derm thickness reaction, are relatively minimal compared with the past type of antivenom. |
|  | Cost | P1:  ... to give somebody a lifesaving medication - It doesn't matter what the cost is, [INT: mhm] it's to save their life. [INT: mhm] So that's why we do what we do. [INT: Right.] So the answer is, I don't care what the cost is, I'm gonna get them to try and get their therapy. [INT: mhm] Luckily, we're not socialized health care where the cost is the limiting factor sometimes. [INT: Right] Here in America, we don't have that. We have, just get the person to care and work out - work out the money later.  INT: Okay. And when you are talking about this treatment with the patient, do you ever bring up the cost of the treatment?  P2: I did, yes. I had a pharmacist look it up.  INT: Ok, and do you think that ever plays into a patient's decision of going forward with the treatment or not?  P2: I think so, yes. It depends on how serious the snake bite is. And if there is an obvious, you know, a risk to limb or life, then the patient's probably less likely to worry about costs. But if it's, you know, kind of one of these questionable snake bites and questionable envenomation, someone's gonna give it to be careful, that may come into factor if it's a couple thousand dollar medicine.  INT: OK, and are you aware of if you get the antivenom from the zoo, does the hospital pay for that or does insurance or where does that come from? P4:I have no idea, honestly.  INT: That's great, thank you. So you mentioned that you would talk about the evidence and some of the benefits and harms. Do you think that anything like cost would come into the conversation?  P6: Yea, I think that if it cost a million dollars for a dose, then probably that's something the healthcare system and the patient will want to know and the insurance company. I think there is probably a threshold above which costs would become an issue. And it's solely would depend on if the patient has to pay out of pocket for that, you can put them into debt for the rest of your life, I don't know what that threshold would be. I would hope that there would be a cost-effectiveness analysis somewhere in the antivenom literature that would help to inform that.  P9: Well, I don't think we can dodge the cost issue.  INT:Uh huh. Can you talk a little more about that?  P9: Well, it's expensive. So, you know, all things being equal, um, it depends who's paying for it. It's not that in a life-threatening situation, you wouldn't make decisions independent of cost. But, you know, given how expensive it is [INT: Mhm] that factors, I mean, that comes up frequently. Like you realize how many vials of antivenom this person has gotten. Do you know how much this cost? [INT: Mhm] You know, people look at their hospital bills and go, oh, my God. [INT: Yeah] You know? And so I think it's it's a cost benefit analysis. [INT: Mhm] And so that's that's a factor. [INT: Yeah] Those are the two biggies.  INT: OK. So who, who usually is paying for the antivenom in your own experience?  P9: It really is, it's like anything else in health care. [OK] Just depends what the patient's insurance coverage is. [INT: Yeah] So, if they have insurance coverage, then hopefully the insurance covers it. But if they don't um and if it's the right thing to do to give it, the hospital has to eat it. I don't think we can hand people uh bills for hundreds of thousands of dollars. Um. And I just don't think we can do that. We’re, you know, [INT: Yeah] That’s across the whole spectrum of health care. [INT: OK] Right. [INT: Right] Well, you know, if you're if you're the company making this stuff, [INT: Mhm] there's a certain profit margin that they I'm sure they expect or would like. Um… But they, you know, compassionate use programs are very important.  INT: Right. Yeah, okay. So do you know how much how much a vial actually is?  P9: I don't know what the cost of that. You know, I don't know what the cost is. [INT: Uh huh] I know that I've seen prices that are in high thousands of dollars per vial. [INT: OK] And I also don't know if there's standard pricing across the country, you know, whether the retail price is the same in northern California as it is in Arkansas.  INT: I see. OK. Great. And then how do you educate the patients um on the cost factor?  P9: You know, um, if I… um if I think the patient is on the bubble about getting antivenom or not – [INT: Mhm] And if I think they're going to get the bill, um, and I think that they should have input into the decision - And this is not just for antivenom, this is for any form of care for which I think the patient should be allowed to have input into whether they get it or not. |
|  | Patient education, shared decision making | INT: Okay, great. So in the, in the two cases that you treated, it sounded like antivenom wasn't even um up for discussion. In, in those two cases. Um. Did you, did you inform the patients at all that, you know, there is antivenom available, and if so, how do you, how do you educate patients on their treatment options?  P5: Interesting. So um when talking about that, I usually phrase it as this looks like this is going to be a more local reaction. And the most important thing I either say they have already done by doing wound care and presenting to the emergency department so they can get checked up because there can be serious complications. So I do that with most of my patients to be like, thank you. You've done 50 percent of the work by just showing- showing up. [INT: Right] And then we'll figure out the rest from here [INT: Mhm] and then inform them about we're going to do some labs. And I want to observe you and I want to let you know- And in case you didn't know, there is a treatment that we give in envenomation in very specific cases when people get really, really sick. [INT: Mhm] Um, your labs and the way you look and feel will tell us how to do that. And then I'm like and I also talk to the poison control people um who are experts in this particular medication. Um. That's sort of how I approach, approach most of the subjects when I talk to them.  P14: Um, I think think for the most part uh, you know, we try I try to cater it based on, you know what I think you know, I don't want to have a conversation I don't think that they they they can understand or truly truly understand what I'm trying to say. So if, you know, if it, so I do try to cater the language and everything to them [INT: mhm], but I think for the most part they do and we're watching them and I, you know, if they want to go ahead with the antivenom, um, and that’s one thing or they're like, well, we'll hold, they understand like if things change, we can always give it then.  INT: And when you're talking to the patient, do you ever bring up the cost into the conversation?  P15: Um, you know, not typically, unless they ask.  INT: OK, great. And how how do you how do you educate or inform the patients about the the possible cost factor, um, when you discuss treatment with them? Does that come up at all? P16: I don't bring it up. [INT: OK] I don't bring it up because, um, uh, I guess I'm afraid that that it might change their opinion, um, either way and stuff on it, you know. I'm not sure if that's a wise approach, um, because, you know, maybe patients should know the prices of stuff when they choose. [INT: Hmm] Um, but I don't, I guess I, I don’t know, I just get a little bit uncomfortable, I don't know how to approach that. And I haven’t figured out the best way to approach, um, like the pharmacoeconomic talk of stuff. So, um, so I haven't. [INT: Yeah, OK], I can’t remember that I've ever brought up the cost. |
|  | Patient behavior and seeking care | P4: I haven't really recommended anti-venom to somebody that has refused it at that point, and I haven't had anybody that insisted upon it that hasn't needed it and then administered it, like there hasn't been that I demand this treatment, but there's no indication for it that we went ahead with, so.  INT: I see. OK. Alright. But from, from your experience, patients are usually pretty um, when they get the recommendation from you they, they usually aren't too too worried about the cost? Or how, how do they usually react to that?  P7: Yeah, so I’ve had people worried about it, but I think that that issue is more just that people realize that like if we've gotten to the point where, ya know, they may really have serious consequences from the snakebite, people seem to be pretty willing to kind of sort it out. |
|  | Treatment hesitancy and perceived confidence | P9: Other people who are more comfortable with clinical judgment, which I think, frankly, tend to be older doctors, because we didn’t have all that stuff [laughs] in those days. So we're used to watching patients.  INT: Yeah. Yeah.  P9: Um… You know, I think so. There's a whole – that, that could be an area of study that you might drive out of this. [INT: Yeah] Which is, you know, depending on what questions you can ask people as are, are older people or people who have been around, more clinically experienced, [INT: Mmm] less likely to give you antivenom.  P12: I would probably have the ED pharmacist or a pharmacist kinda go over with them uhm, that uhm, or I would you know, really have to review the package insert before I talk to the patient, or an article that is update [INT: mmmh] as a reference and review it. Just because again, for something that is so so so infrequent, [INT: yeah] uhh, I think it would be important to take a reference or just to be sure I am uh, providing them all the accurate best information.  INT: OK. And do you think that, you know, you mentioned that you stay current with the literature, do you think that besides reading journals, there's a way to, you know, get this information out if people aren't aware of the current literature? Or do you think that just, you know, relying on calling the poison center is how physicians typically get their information if they're not an expert in this area?  P14: Yeah, I mean, I think there's ways to get out, uh, what they're webinars or other types of educational opportunities to get things out. Um, with that said, I think for someone who's only going to deal with maybe a couple a year, um, I think they're always going to call for help to make sure they're doing the right thing [INT: right]. And, even if they're reading something , there is a difference between reading about something and and truly seeing it enough that you feel comfortable  INT: OK. So you can't think of any reason why they wouldn't  [administer antivenom]?  P2: I mean, maybe just like I said, maybe uncomfortableness with delivering [fab antivenom] and treating snake bites because of the limited amount that we have here in Orlando.  INT: Do you feel that there's any hesitation among physicians to use antivenom?  P4: Not that I've experienced, no.  P8: And uhm, so you know everyone gets excited, everybody wants to give [fab antivenom] because the rep was there a couple of months ago and [fab antivenom] was introduced, and I said, let’s just wash the wound off and swab the area, look at it, mark it, elevate his arm and I wasn’t too convinced of using any [fab antivenom]. Because I think when you want to use [fab antivenom], you have to be very cautious before you make that decision now.  P9: Well, you only get confident by practicing. [INT: Uh huh] So, yeah, you know, that part's gonna be a little tougher. [INT: Right] |
| **Resources** | Usage and perceptions on available resources | INT: Do you know of any other information resources that you can tap in, tap into ?  P1: Yes , we have uh -- I have -- We have online resources. The X health system is very robust with information [INT: mhmm] and data. We can tap into an online toxicology…. online… wilderness medicine just so we can identify the bite marks. [INT: OK .] There's a huge library of data that we could access. whatever, whatever you think you want to look for [INT: Yeah], we just log in and look for it.  P2: Yeah, I mean I think it would be based on, in our facility it is based on the physicians. I mean, obviously the patients have to agree to do it with some side effects that can attract with allergic reactions. But yeah, we don't have a protocol for it. We would probably use Poison Control's input as well. Most physicians I think would, but it's still the physician's decision, yea.  INT: OK, so you talked a little bit about the policies include calling poison control if you need consultation. Are there any other policies at your current workplace in terms of snake bite treatments?  P2:  I wouldn´t say it´s a policy to call poison control, I think it is a thing most physicians would do, due to you know due to the limited amount of snakebites that we receive. But we actually don’t have any policy written. So, it would be individually based on the physician. INT: OK. And do you think that there's a common uhm, Physicians would tend to treat snake bite patients in the same way at your facility or there might be…are there any different opinions on how to treat them? P2: I would imagine there is different opinions. Without a protocol, there´s always different opinions.  INT: Where do those resources come from that guide your decision making? P3: Yeah. Umm… our um management. Yes, we do- we do have a champion. And that would be where bias in the sense that Dr.A is co-investigator on all these snakebite studies. [INT: Mhm] But our current document literally is a cut and paste, comes from the article by Lavonas et al, 2011, currently “Unified treatment algorithm for the management of crotaline snakebite in the United States: results of an evidence-informed consensus workshop”. And we have their- their, uh, flow chart. [INT: OK] And that's why I'm looking at and that's that's what I would pull from – and I think is similar to what the poison center has. They may have some updated stuff, but [INT: Mhm] but this is 2011, so.  P05: And and uh. Yeah, and then just looking it up when seeing those two patients [INT: OK] going to Up-To-Date or some other E.M. reference guide, Rebel EM, EMcrit, EMrap are some of the specific sites that I use, um then uh then I would have to say that would be it in total. |
|  | The role of scientific evidence | P6: So I'd be looking for I think reasonable levels of evidence showing benefits outweigh harms in the forms of systematic reviews of observational studies, again using the Bradford Hill criteria (INT: Mhm) and then single observation studies would be below that. I would not accept a single patient case report.  INT:  OK, thank you, that's very informative. OK. So you'd be looking for evidence to show that it is supported that the benefits outweigh the harms. So if there was no evidence that antivenom, no strong evidence, maybe there is questionable evidence- how do you think that would play into your decision making?  P6: I think that if the toxicology team was recommending an antivenom therapy and there wasn't any evidence to support it or very weak evidence or contradictory evidence where some evidence suggest benefits, some evidence suggests harms, and some evidence suggest both, I would, with the toxicologist want to go into patient's room and discuss what we know about the antivenom therapy, the potential benefits, potential harms and then in a shared decision making way come to a decision. The toxicology, myself and the patient about the best option for that patient.  INT: Okay. I got you. Great. So, what do you think would need to, what do you-think we would have to do, and, and, research to get to that point? What would you suggest? What kind of date would -  P13: Um, I think you would. Um, I think you would have to have a very a very standardized dosing regimen, timing regiment, and um, specific criteria for what you’re using as mild, moderate, serve, um, to show benefit and then I think that on top of that, you’d have to argue what does one person consider “back to function”, right? That’s where the subjectivity of it comes in right like? [INT: Yeah] Like I’m two weeks out on a patient or like, you know, do they feel like they have 90 percent of their hand back or 75 percent of their hand back? Like, that’s going to be a very hard depiction, you know [INT: hmm] unless you find some objective criteria like grip strength or, um, you know some, some specific objective criteria they can do in the ED- it’s not a, it’s not a ‘I feel this way’ it’s a ‘I could do this activity’ or ‘I could do this activity’ which would make me therefore seem like I’m back to my function.  INT: Yeah, OK, great. And how how do you yourself stay up to date with recent literature or best practices when it comes to snake bite treatment? [P16: Ummm] Where do you get your information?  P16: I think I do end up reading all the paper, all the snake related papers in the, um, in the emergency medicine literature. [INT: Mm hmm]. Umm, yeah, I think I would be pretty familiar with most of those, um, recently went to umm, a venom week [INT: Mhm] conference, that was, uhh, I don’t know, last-weekish maybe? Umm, yeah, so I’ve been to those a few times. |
| **Suggestions** | Information dissemination, Guidelines, Feedback | INT: So is there any suggestions that you have on how you think snake by patient treatment can improve locally or globally?  P10: So definitely two very different questions. [INT: OK] So, you know, I think locally it has to do with more so with the whole health care system. Right. So if you're going to charge me twenty thousand dollars per vial of antivenom, that is very beneficial to them um, that’s a big problem, right. [INT: Mhm] Um. Globally, the use of antivenom potentially costs, you know, 20, 30 dollars, a hundred dollars per vial. I understand that there's need for innovation, and drug development costs that goes into it. So I think it's a huge systemwide problem that this orphan drug costs so much money. I think having greater availability of antivenom at lower cost can take the question of whether or not to give antivenom to patients, in the equation that anyone with minimal symptoms may be able to get the antivenom without just thinking about how it will affect them financially.  INT: So if you know if there were literature, basically with similar set up that was done for adults, in terms of length of hospital stay, recovery time, all that stuff, would you expect it to be different for the pediatric population?  P11:  Uh I don’t know uh it’s a good question, I don’t know, I would uh I would expect uh probably not uh you know and again, like I guess the question is like what markers are you looking for, like return to function, uh like because obviously kids aren’t going back to work, but like going back to school and you know participating in their activities and sports, [INT: Yeah] so maybe the markers would be a little different, I don’t know that I would expect a tremendous difference, but I mean again that another reason to study it because maybe there is.  INT: Yeah right.  P11: Again, I don’t know that people are going to do it, because it a lot of times stuff like this it’s not, it’s harder to study stuff like this for kids for various reasons.  INT: OK, or maybe any suggestions for how physicians can be better informed about this or anything that would be helpful if you did have a snakebite patient that would help you in treating the patient better?  P6: No, I think my lack of exposure to these patients limits my ability to answer that question.  P5: My thought at this point in time that if there is a… if it comes back and from the manuscripts, a conclusion that could be made, that there is quite a variable at this, because this is sort of a low frequency but potentially highly acute event um that that tells me there needs to be more education, but not only in, not only education, but also as you're mentioning, and I hope we can get to at some point in time, a standard or a guideline that we can all appreciate. [INT: Mhm] And then following that, the only thing that if I ask for, if I ask for a guideline, the important part is how would it be disseminated and implemented? [INT: Mhm] And finally how would it how would it be tracked to make sure that people can follow this guideline in their practice areas and then act? And then the last part of that being, if people aren't following practice guidelines, um, what are the barriers um to prevent them from doing that? And hopefully doing that in a very non-confrontational way, which seems to be nearly impossible in the world of quality assurance and [INT laughs] and risk management in our current practice. Um… Meaning that this is about this is about education and elevating everybody's standard of care, um, making it about the patients and our communities to make sure they're doing the right thing. [INT: Mhm] Um. But so we're sort of being socially and fiscally responsible with which I'm guessing is a pretty expensive medication. I'll venture to guess, is it like five thousand per dose that we give? |
