# Supplementary material for: BITES study: A qualitative analysis among emergency medicine physicians on snake envenomation management practices
Source: PLoS One. 2022 Jan 7;17(1):e0262215. doi: 10.1371/journal.pone.0262215 (PMC8741014; doi:10.1371/journal.pone.0262215)
Supplement: S1 Scheme — (DOCX) [file pone.0262215.s003.docx]

| Selective code | Axial code | Open code |
| --- | --- | --- |
| Snake Bite treatment approach, clinical decision making | Alternative, supplemental treatment options | supportive care for dry bites; infection control; elevation; vaccination status; surgical debridement; pain management; medications; follow-up care; rehabilitation services; contra-indications; observation |
|  | Commonalities among snake bite patients | different by state and snake species; seasonal; setting |
|  | Patient assessment | may start remotely by expert; clinical assessment parameters; area of bite; clinical tests ordered; adults vs pediatrics; clinical course, possible hospital admission |
|  | Patient behavior, care seeking behavior | cultural implications; geography and access to care impacts behavior; need for public health education on treatment; willingness to follow MD advice |
|  | Patient education/ shared decision making | available treatment options; influence of symptoms; dry bites; varying levels of education provided; treatment suggestion; education on first aid; cost of antivenom to the patient; type of snake and expected symptoms; need for more data on cost, feedback loop of outcomes to inform shared decision making; patient friendly language |
|  | Providing education on snake bites to trainees | continuing education; levels of education during training (medical school, residency); exposure; local experts; role of toxicologists |
|  | Referral out to different facility for management | exotic snakes; available resources; awareness of possible referral locations |
|  | Snake bite example | recollection of specific snakebite patients treated |
|  | Snake identification | venomous vs non-venomous; usage of different resources to identify species; importance of snake identification for clinical management; referral system depending on species; images of snake provided by patients to identify |
| Antivenom treatment | Availability, accessibility | potential barrier; storage of antivenom; pharmacist's role; depends on institution, network, location; exotic snakes challenge; delivery from other institutions; awareness of available snakebite treatment centers |
|  | Cost transparency, coverage | influencing factor when not live saving; cost-benefit analysis; awareness of costs to patients; role of insurance; differences between hospitals; education on cost; reduce numbers of vials to reduce costs |
|  | Dosage | based on treatment algorithms/ guidelines; snake species; time frame to administer antivenom |
|  | Effectiveness and indications | no contraindication to overtreat/ treating early; risk of loss of life or limb if untreated; number of bites, location of bites, number of joints involved; perceived dysfunction; not indicated for dry bites; depends on snake species; snakebite induced compartment syndrome; reliance on experts to determine indication; lab abnormalities; systemic involvement; coagulopathy; shock; role and indication of maintenance vials; pain alone might not be an indication; potential to reduce opioid use; age; pediatric population; time to return to functional activities; possible positive impact on swelling, pain and recovery time; prolonged envenomation syndrome; expected recovery; impact on long term health |
|  | Risks/ side effects | allergic reactions; serum sickness; low risk; perceived safety; interactions with other medications; potentially increased risk in people with certain conditions and allergies; cost |
|  | Treatment hesitancy/ Perceived competence in making clinical decision | lack of perceived competence to choose dosage; influence of training and prior education; influence of physician's personal approach/ beliefs to treat; cost; lack of available follow up data, effectiveness on long-term health outcomes; depends on snake species and severity of bite, risk of mortality; clinical indication not always present; role of poison control; experience with previous forms of antivenom and more severe side effects; lack of access could lead to hesitancy to treat with antivenom |
| Resources | Poison Control | snake identification, clinical assessment, lab values, observation times, anti-venom recommendation/dosage, nonspecific for pediatric, and to referral to nearby institutions with snake management expertise; reconfirm treatment suggestion; available 24/7 over the phone; potentially at bedside; high quality information; public health monitoring |
|  | Quality of available resources | different levels of quality; information snake species specific, cannot always be transferred; limited evidence |
|  | Local experts | within own institution or poison control; personal connections with experts for guidance; herpetologist society |
|  | Miscellaneous | own clinical experience and judgment may take precedence over available resources in clinical decision making; zoo for specific species; scientific literature; pharmacist |
|  | Relying on previous education on Snake bites | self-driven interest to learn about available evidence; mentorship; formal education; trained in high prevalence areas |
|  | Treatment Guidelines | policy, institutional guidelines; published scientific literature on treatment algorithm; physical copies or online resources; awareness of available guidelines; manufacturers and FDA approved guidelines/ package insert for dosage |
|  | Online and textbooks | provided by institution; wilderness medicine library; chosen by physicians individually to access resources; apps; websites; blogs; standard literature |
|  | Toxicologists | on-call or at bedside; variations in expertise for snakebite management |
| Scientific evidence |  | suggestions/ need for additional evidence; robust designs; lack of awareness of high-quality studies; high quality data needed to justify cost; level of trust in scientific data; pediatric specific data |
| Suggestions |  | education; information dissemination; cost innovation; shared decision making; protocols; assess level of competence/ knowledge; awareness on accessing high quality information |
| Participant, Background, Institution | Current facility | Table 1 information |
|  | Formal Education on snake bite management |  |
|  | Participant Demographics |  |
|  | Personal Experience treating snake bites |  |
|  | Professional background |  |
